# Attention‐deficit/hyperactivity disorder is associated with increased risk of cardiovascular diseases: A systematic review and meta‐analysis

**DOI:** 10.1002/jcv2.12158

**Published:** 2023-04-05

**Authors:** Lin Li, Honghui Yao, Le Zhang, Miguel Garcia‐Argibay, Ebba Du Rietz, Isabell Brikell, Marco Solmi, Samuele Cortese, J. Antoni Ramos‐Quiroga, Marta Ribasés, Zheng Chang, Henrik Larsson

**Affiliations:** ^1^ School of Medical Sciences Örebro University Örebro Sweden; ^2^ Department of Medical Epidemiology and Biostatistics Karolinska Institutet Stockholm Sweden; ^3^ Department of Biomedicine Aarhus University Aarhus Denmark; ^4^ Department of Psychiatry University of Ottawa Ottawa Ontario Canada; ^5^ Department of Mental Health The Ottawa Hospital Ottawa Ontario Canada; ^6^ Ottawa Hospital Research Institute (OHRI) Clinical Epidemiology Program University of Ottawa Ottawa Ontario Canada; ^7^ Department of Child and Adolescent Psychiatry Charité Universitätsmedizin Berlin Germany; ^8^ Centre for Innovation in Mental Health‐Developmental Lab School of Psychology University of Southampton Southampton UK; ^9^ Solent NHS Trust Southampton UK; ^10^ Hassenfeld Children's Hospital at NYU Langone New York University Child Study Center New York Hampshire USA; ^11^ Division of Psychiatry and Applied Psychology School of Medicine University of Nottingham Nottingham UK; ^12^ Department of Psychiatry and Forensic Medicine Universitat Autònoma de Barcelona Barcelona Spain; ^13^ Biomedical Network Research Centre on Mental Health (CIBERSAM) Madrid Spain; ^14^ Department of Mental Health Hospital Universitari Vall d'Hebron Barcelona Spain; ^15^ Psychiatric Genetics Unit Group of Psychiatry Mental Health and Addiction Vall d’Hebron Research Institute (VHIR) Universitat Autònoma de Barcelona Barcelona Spain; ^16^ Department of Genetics, Microbiology, and Statistics Faculty of Biology Universitat de Barcelona Barcelona Spain

**Keywords:** attention‐deficit/hyperactivity disorder, cardiovascular diseases, meta‐analysis, observational studies, systematic review

## Abstract

Attention‐deficit/hyperactivity disorder (ADHD) often co‐occurs with other psychiatric and physical diseases. However, available evidence on associations between ADHD and cardiovascular diseases (CVDs) is mixed. To systematically review, quantitatively synthesize, and appraise available evidence on the link between ADHD with CVDs, we searched relevant articles in PubMed, Embase, PsycINFO, and Web of Science from inception to May 1, 2022. Study quality was assessed by using the Newcastle‐Ottawa Scale, and random‐effects model meta‐analyses were performed. A total of 18,391,169 (ADHD: *n* = 421,224) individuals from 11 studies were included in our systematic review and 8,196,648 (ADHD = 332,619) individuals from five studies were included in the main meta‐analysis of adjusted estimates. Pooled estimates showed that ADHD was significantly associated with an increased risk of CVDs in analyses based on adjusted effect size (odds ratio (OR) = 1.96; 95% confidence interval (CI) = 1.19–2.23, *Q* = 140.74, *P*_Q_ < 0.001, *I* ^2^ = 97.2%). When restricted among adults, the heterogeneity declined to null (OR = 1.73; 95% CI = 1.14–2.62, *Q* = 6.28, *P*_Q_ = 0.10, *I* ^2^ = 6.28%), suggesting age might be the main source of heterogeneity. In subgroup analyses, we found increased risk of CVDs associated with ADHD across age groups, type of CVDs, and data sources. This systematic review and meta‐analyses indicate that ADHD is associated with increased risk for CVDs, but further studies with various study designs are warranted to advance the understanding of the underlying mechanisms for the observed association between ADHD and CVDs. Additional research is also needed to resolve the role of ADHD medications which remains unclear due to the limited number of primary studies exploring this issue.

## Graphical abstract

Individuals with ADHD were nearly twice as likely to develop at least one CVD, compare with non‐ADHD. The observed strength of associations were largely comparable to estimates of associations between sever mental illness and CVDs.

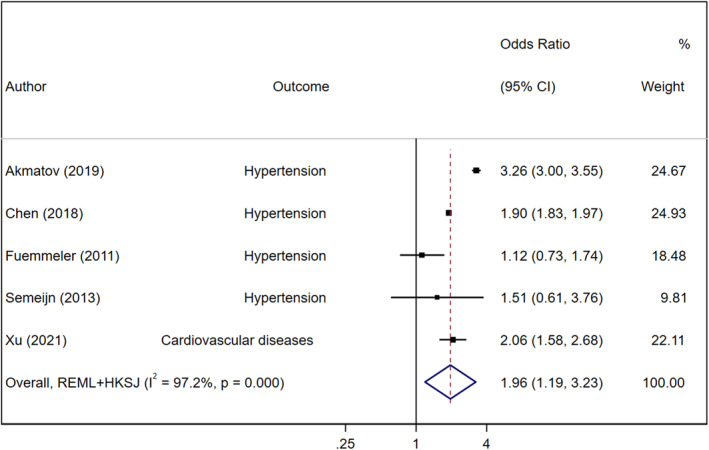

**Key Points**

- Evidence on the associations between ADHD and CVDs is mixed.
- Individuals with ADHD were nearly twice as likely to develop at least one CVD, compare with non‐ADHD.
- The observed strength of associations was largely comparable to estimates of associations between severe mental illness and CVDs.
- More studies are needed to explore the role of ADHD medications.

## INTRODUCTION

Cardiovascular diseases (CVDs) are the leading cause of morbidity, mortality, and rising health care costs worldwide (Mensah et al., 2019; Vos et al., 2020). Cardiovascular diseases caused an estimated 18.6 million deaths in 2019 worldwide, corresponding to almost 400 million years of life lost and another 34.4 million years lived with disability (Roth et al., 2020). It is therefore a public health priority to gain further insight into the factors that contribute to CVDs in order to guide prevention and clinical practice. Besides traditional CVD risk factors, such as overweight/obesity, diabetes, and smoking, concerns around the risk for CVDs in individuals with psychiatric disorders are growing (Correll et al., 2017, 2022; Everson‐Rose & Lewis, 2005; Pedersen et al., 2017; Solmi et al., 2021).

Attention‐deficit/hyperactivity disorder (ADHD) is one of the most prevalent psychiatric disorders, affecting around 5% of children and 2.5% of adults (Davidson, 2007; Faraone et al., 2015). It is characterized by developmentally inappropriate, pervasive and impairing inattention and/or hyperactivity‐impulsivity. In addition to the core clinical symptoms of ADHD, co‐occurring poor mental and physical comorbidities are prevalent in individuals with ADHD (Angold et al., 1999). However, compared with the extensive research of psychiatric comorbidities in ADHD (Gnanavel et al., 2019), physical comorbidities (Arrondo et al., 2022), such as CVDs, have received less attention, particularly among adults (Instanes et al., 2018).

There are several reasons why individuals with ADHD could be at increased risk for CVDs. First, previous research suggests that the link between ADHD and CVDs is biologically plausible via immune system abnormalities (Fernández‐Ruiz, 2016; Hoekstra, 2019), neuromodulator dysregulation (Ahmad Banday & Lokhandwala, 2006; Misener et al., 2004), and dysregulation of the hypothalamic‐pituitary‐adrenal (HPA) axis (Corominas et al., 2012; Jokinen & Nordström, 2009). Second, individuals with ADHD are at increased risk for unhealthy lifestyle factors (e.g., smoking, obesity, poor physical activity) (Cook et al., 2014; Cortese et al., 2015; van Amsterdam et al., 2018), which are all well‐established risk factors for CVDs (Dwivedi et al., 2020; Mons et al., 2015; Wilmot et al., 2012). Third, previous studies indicate that several psychiatric comorbidities of ADHD, for example, depression (Hare et al., 2014; Nicholson et al., 2006), substance use disorders (Aldridge et al., 2018; Roerecke & Rehm, 2014), schizophrenia (Hennekens et al., 2005), bipolar disorder (Swartz & Fagiolini, 2012), and anxiety disorder (Tully et al., 2016), are associated with CVDs. Fourth, even though findings are inconclusive, there has been a long‐standing concern around a potential increased risk of cardiovascular events due to the use of stimulant medications to treat ADHD (Liu et al., 2019; Zhang et al., 2022). However, the available epidemiological findings are mixed, despite the potential hypotheses for an increased risk of CVDs in ADHD.

The aim of the current study was to conduct a systematic review and meta‐analysis to provide a quantitative summary of observational studies on the associations between ADHD and CVDs. A second aim was to evaluate the impact of study design and confounder adjustment (e.g., sociodemographic factors, traditional risk factors for CVDs, and use of medications) on the observed associations, in order to provide insights into underlying mechanisms.

## METHODS

The study was conducted and reported following the Preferred Reporting Items for Systematic Reviews and Meta‐Analyses guidelines (Boutron et al., 2021) and Conducting Systematic Reviews and Meta‐Analyses of Observational Studies of Etiology (Dekkers et al., 2019). This protocol was registered in the International Prospective Register of Systematic Reviews (PROSPERO: CRD42021274367) (Lin Li et al., 2021).

### Search strategy

A systematic search for observational studies was conducted in PubMed, Embase, PsycINFO, and Web of Science databases, up to May 1, 2021, with no language and article type restrictions. We used various combinations of the following keywords “cardiovascular disease”, “coronary heart disease”, “heart disease”, “sudden death”, “ischemic heart disease”, “hypertension”, “cerebrovascular disease”, “stroke”, “transient ischemic attack”, “attention‐deficit hyperactivity disorder”, “central nervous system stimulants” and “observational study”. The complete search strategy is presented in Table S1. In addition, we performed manual searches through the reference lists of original publications and reviewed articles to identify further pertinent studies.

### Inclusion criteria

We included all types of observational (cross‐sectional or prospective) studies providing data on the strength of the association between ADHD and CVDs in children, adolescents, or adults. Eligible definitions of ADHD were as follows: (a) a categorical diagnosis of ADHD according to the DSM‐III, DSM‐III‐R, DSM‐IV, DSM‐IV‐TR, DSM‐5, ICD‐9, or ICD‐10; (b) ADHD‐medication prescriptions as a proxy for ADHD diagnosis; (c) ADHD symptoms based on value above cut‐off on a validated self/parents/teacher‐reported ADHD questionnaire; (d) ADHD diagnosed via a structured psychiatric interview or positive answer by self/parents/teachers to the question ‘Did your doctor ever tell you that you/the child have ADHD?’ Titles, abstracts, and full text of included studies were screened independently by two authors. Discrepancies were resolved through discussion with a senior investigator.

The main outcome was the maximally adjusted odds ratio (OR), risk ratio, and hazard ratio, with their corresponding 95% confidence interval (CI) ratio expressing the association between ADHD and CVDs. The secondary outcome was the unadjusted estimate of the associations. When sufficient data (i.e., sample size, prevalence of ADHD, CVDs) was available, crude ORs were manually calculated or obtained by contacting the original authors, if not reported in the original paper. The choice of primary and secondary outcomes was made because adjusted estimates are more informative and potentially less prone to biased derived from the selection of the participants (Cortese et al., 2022).

### Data extraction

The following information was extracted from each study for the qualitative and quantitative synthesis: name of the first author, year of publication, sample size, data source, study country, age and sex for participants, study design, years of original data collection, the definition of ADHD and CVDs, effect size (ORs, RRs and HRs) and information on confounding adjustment. We extracted both adjusted and unadjusted ratios if available, and we used the maximally adjusted ratios in the analyses. Unadjusted effect sizes were calculated based on the information provided in the paper when necessary. Two authors conducted the data extraction process separately, and any disagreements were resolved by discussing with a third investigator.

The quality assessment of eligible studies was performed using the Newcastle‐Ottawa Scale (NOS) by two independent authors, and the discrepancies were solved by consensus. This 9‐star scale consists of three parts: selection of participants and the measurement of exposure (4 stars), comparability (2 stars), and assessment of outcomes and adequate follow‐up (3 stars) (Wells et al., 2000). A higher score on the NOS represents a higher‐quality study, and generally, a score of 0–3, 4–6, or 7–9 is regarded as low‐, moderate‐, or high‐quality, respectively (Wells et al., 2000).

### Statistical analysis

We first described the characteristics of the included studies and various confounding adjustment strategies to provide an overall picture of the current evidence. Meta‐analyses were conducted using random‐effects models in order to take into account heterogeneity between studies, and the results were summarized in forest plots. When there were several studies from the same population, only the one with the largest sample size was included in the meta‐analysis to avoid overrepresentation bias (Li et al., 2020). ORs from logistic regression and HRs from Cox regression were combined because they closely approximate each other (D'Agostino et al., 1990). We then meta‐analyzed adjusted and unadjusted ORs across all studies. Additionally, several subgroups and sensitivity analyses were conducted to investigate whether the main results were robust across age groups (adults and children separately), specific types of CVD (hypertension and other types of CVDs), and different data sources (registers and research samples).

Heterogeneity across studies was described by Cochran's Q test and the inconsistency index (I^2^). If significant heterogeneity was detected by the Q test, we considered I^2^ values greater than 75% as high heterogeneity (Higgins & Thompson, 2002). Restricted maximum likelihood method was used to estimate between‐study variability, with Hartung‐Knapp‐Sidik‐Jonkman CI for the summary effect. To evaluate each study's effect on the overall effect size, a leave‐one‐out analysis was also conducted. The publication bias was first assessed through visual inspection of the funnel plot and then tested quantitatively with Egger's test. All analyses were performed with Stata 16.0 (StataCorp L.P., College Station, TX).

## RESULTS

### Study characteristics

**FIGURE 1:**
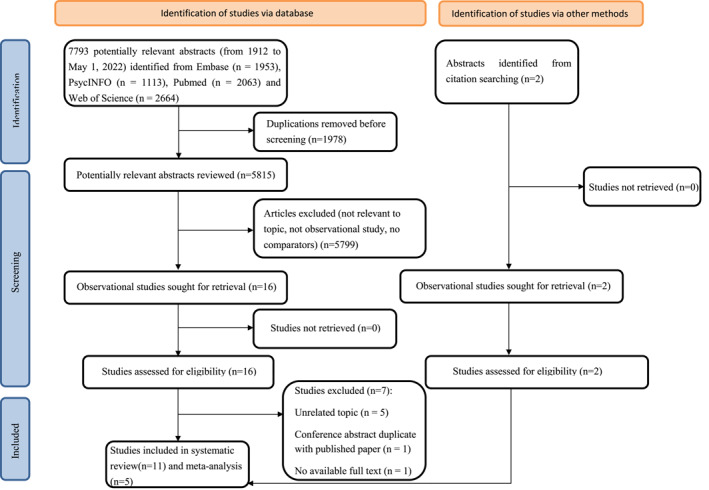
Preferred Reporting Items for Systematic Reviews and Meta‐Analyses (PRISMA) flow diagram for inclusion of the studies examining the association between Attention‐deficit/hyperactivity disorder (ADHD) and cardiovascular diseases (CVDs).

The study selection process is shown in Figure 1, and the list of excluded articles (with reasons) after the full‐text screen is presented in Table S2. Table 1 shows the main characteristics of the 11 original studies included in the systematic review (Akmatov et al., 2019; Chen et al., 2018; Du Rietz et al., 2021; Fuemmeler et al., 2011; Grisaru et al., 2018; Li et al., 2022; Nilgün et al., 2018; Ramos Olazagasti et al., 2013; Semeijn et al., 2013; Spencer et al., 2014; Xu et al., 2022). Among the 18,391,169 participants from six countries, a total of 421,214 individuals were with ADHD. The age of participants ranged from 5 to 81 years old, and 9,889,671 (53.8%) participants were men. The publication years were between 2011 and 2022. The most common study design included in the meta‐analysis were cross‐sectional (*n* = 4) (Akmatov et al., 2019; Chen et al., 2018; Semeijn et al., 2013; Xu et al., 2022), cohort (*n* = 4) (Du Rietz et al., 2021; Fuemmeler et al., 2011; Li et al., 2022; Ramos Olazagasti et al., 2013), followed by case‐control (*n* = 3) (Grisaru et al., 2018; Nilgün et al., 2018; Spencer et al., 2014) studies. These studies were conducted in the United States, Sweden, Germany, Turkey, Netherlands, and Canada. Of the 11 studies, five (Akmatov et al., 2019; Chen et al., 2018; Du Rietz et al., 2021; Fuemmeler et al., 2011; Li et al., 2022) obtained data from healthcare/insurance registries, five (Grisaru et al., 2018; Nilgün et al., 2018; Ramos Olazagasti et al., 2013; Semeijn et al., 2013; Spencer et al., 2014) obtained data from research samples, and one (Xu et al., 2022) used data from a specific national cohort. Therefore, register‐based or electronic health care databases comprising large numbers of participants were the most commonly used data source for the studied associations. The definition of ADHD varied across studies, self/teacher/parent‐reported ADHD symptoms (Fuemmeler et al., 2011; Ramos Olazagasti et al., 2013; Semeijn et al., 2013; Spencer et al., 2014; Xu et al., 2022) were the most widely used measurements, followed by electronic health records based on codes in the ICD codes or the DSM‐IV (Akmatov et al., 2019; Chen et al., 2018; Du Rietz et al., 2021; Li et al., 2022; Nilgün et al., 2018). With regards to the CVD outcomes, various measurements were used, including CVD diagnosis based on ICD codes and several specific recommendations/guidance for hypertension (e.g. Recommendations of the American Heart Association). The most commonly studied CVD outcomes were hypertension (Akmatov et al., 2019; Chen et al., 2018; Du Rietz et al., 2021; Fuemmeler et al., 2011; Grisaru et al., 2018; Nilgün et al., 2018) and any type of CVDs (Li et al., 2022; Ramos Olazagasti et al., 2013; Semeijn et al., 2013; Spencer et al., 2014; Xu et al., 2022).

**TABLE 1:** Overview of studies included in the systematic review.

Study	Country	Data sources and year of original data collection	Study design	Sample size	N (CVD)/N (ADHD)	N (CVD)/N(NON‐ADHD)	Male (%)	Mean age or age range	Exposure		Outcomes		NOS
									ADHD definition	Age at assessment	Definition	Diseases	
Akmatov (2019)^a^	Germany	Insurance claims data, 2017	Cross‐sectional	2,586,620	4837/258,662	16,519/2,327,958	75.6	5–14	ICD‐10	5–14	ICD‐10	Hypertension	7
Chen (2018)^a^	Sweden	National medical registers, 1968–2013	Cross‐sectional	5,551,807	2072/61,129	247,870/5,490,678	50.8	40.55 ± 13.49; 18–64	ICD‐9 and 10	18–64	ICD‐8, 9 and 10	Hypertension	6
Du Rietz (2021)	Sweden	National medical registers, 1932–2013	Cohort (follow for 47 years)	4,789,799	NA/61,960	NA/4,727,839	51.0	18–81	ICD‐9 and 10, ADHD medication prescription	N/A	ICD‐8, 9 and 10	Hypertension, ischaemic heart disease, pulmonary disease, atrial fibrillation, heart failure, stroke, peripheral vascular disease	8
Fuemmeler (2011)^a^	United States	National longitudinal study of adolescent health, 1995–2009	Cohort (follow for 33 years)	11,015	46/262	NA/10,753	51.0	28.80 ± 0.12	Self‐report	N/A	7th report of the joint national committee on prevention detection, evaluation, and treatment of high blood pressure: SBP ≥160 mm Hg and DBP ≥100	Hypertension	7
Grisaru (2018)	Canada	Research sample, 2007–2013	Case‐control	3804	3/55	53/3749	51.1	6–19	Medical record	6–19	The fourth report on the diagnosis evaluation and treatment of high blood pressure in children and adolescents	Hypertension	2
Li (2022)	Sweden	National medical registers, 1941–2013	Cohort (follow 11.8 years)	5,389,519	663/37,027	62,089/5,352,492	48.9	38.44 ± 12.32; 18–73	ICD‐9 and 10	3–73	ICD‐8, 9 and 10	CVD	9
Nilgün (2019)	Turkey	Research sample, 2012	Case‐control	177	10/77	9/100	24.3	8.95 ± 2.68; 5–15	DSM‐IV	N/A	Recommendations of the American heart association	Hypertension	3
Olazagasti (2013)	United States	Research sample	Cohort	271	37/135	37/136	100.0	41.4 ± 2.9	Teacher and parent rating	41.4 ± 2.9	N/A	CVD	3
Semeijn (2013)^a^	Netherlands	Research sample, 2008–2009	Cross‐sectional	231	7/23	67/208	40.7	71.6 ± 7.7	Semi‐structured diagnostic interview	N/A	Self‐report	CVD	5
Spencer (2014)	United States	Research sample	Case‐control	198	1/98	1/100	45.5	31 ± 11	The adult ADHD self‐reportscale (ASRS) v1.1 symptom checklist	N/A	Self‐report	Heart attack	5
Xu (2021)^a^	United States	National health interview survey, 2007 and 2012	Cross‐sectional	57,728	256/1790	7650/55,938	47.7	18 or older	Self‐report	N/A	Self‐report	All CVD	5
												Coronary heart disease	
												Stroke	

^a^ Included in the adjusted meta‐analysis.

**FIGURE 2:**
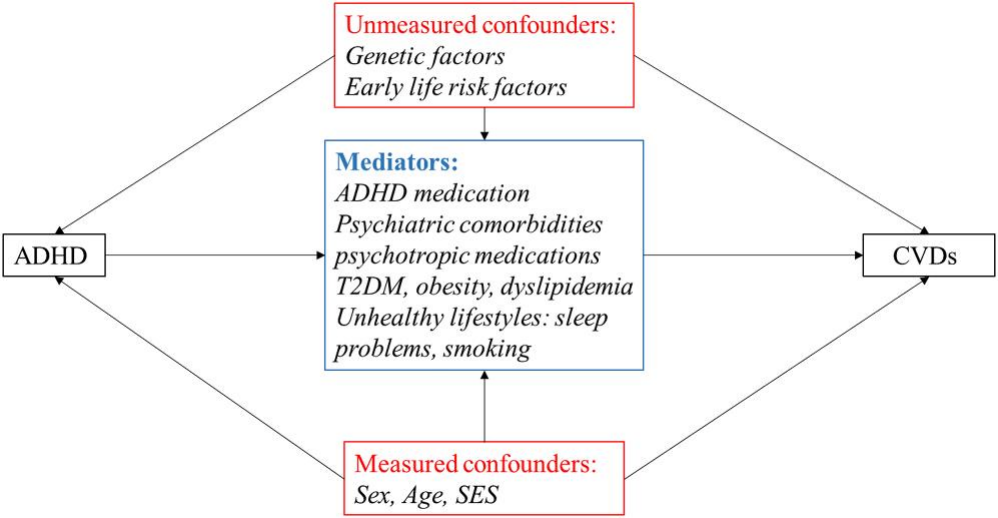
Casual diagram representing the potential pathways of the association Attention‐deficit/hyperactivity disorder (ADHD) and cardiovascular diseases (CVDs).

As shown in Table 2, seven (Akmatov et al., 2019; Chen et al., 2018; Du Rietz et al., 2021; Fuemmeler et al., 2011; Li et al., 2022; Semeijn et al., 2013; Xu et al., 2022) studies (64%) accounted for potential confounders by adjusting for a number of measured covariates. However, inadequate adjustment for confounding was commonly found among included studies. Three studies (Chen et al., 2018; Du Rietz et al., 2021; Semeijn et al., 2013) only adjusted for age and gender, while the other three studies (Fuemmeler et al., 2011; Li et al., 2022; Xu et al., 2022) also included sociodemographic characteristics (e.g., ethnicity, education), psychiatric comorbidities, lifestyle factors (e.g., smoking, alcohol use, and physical activity), and metabolic conditions (e.g., obesity, type 2 diabetes, and hyperlipidemia). However, these lifestyle factors, psychiatric comorbidities, and metabolic conditions are more likely mediators in the pathways linking ADHD to CVDs (Figure 2) rather than confounders. The potential role of familial factors was only explored in one Swedish study (Du Rietz et al., 2021). Of particular importance, only one study examined the contribution of ADHD medication to the association between ADHD and CVDs in a sensitivity analysis, and suggested null effect of ADHD medication (Li et al., 2022).

**TABLE 2:** Adjusted covariates in studies of Attention‐deficit/hyperactivity disorder (ADHD) and cardiovascular diseases (CVDs).

				Covariates adjusted in each included study										
Study	Gender	Age/Year of birth	Region/race/ethnicity	Education achieved	Metabolic conditions^a^	Family income	Depression	Other psychiatric comorbidities^b^	Smoking	Alcohol	Psychiatric medications	Family history of CVD	Physical activity	BMI
Akmatov (2019)	×	×	×											
Chen (2018)	×	×												
Du Rietz (2021)	×	×												
Fuemmeler (2011)	×	×	×	×			×		×	×			×	×
Grisaru (2018)	N/A													
Li (2022)	×	×	×	×	×		×	×	×		×	×		
Nilgün (2019)	N/A													
Olazagasti (2013)	N/A													
Semeijn (2013)	×	×												
Spencer (2014)	N/A													
Xu (2021)	×	×	×	×		×			×	×				

^a^ Metabolic conditions include obesity, type 2 diabetes, dyslipidemia.

^b^ Other psychiatric comorbidities include anxiety disorder, autism spectrum disorder, bipolar disorder, conduct disorder, depressive disorder, eating disorder, intellectual disability, personality disorder, schizophrenia and substance use disorder.

The quality scores based on the NOS ranged from 2 to 9 stars (Median: 5), suggesting an overall moderate quality of the included studies. As shown in Table S3, the number of stars represented the score of each item. Generally, most studies used well‐defined exposures and outcomes, but some included studies with one or no star in relation to ‘Comparability’ did not adjust for sex, age, and other sociodemographic characteristics.

### Meta‐analysis

**FIGURE 3:**
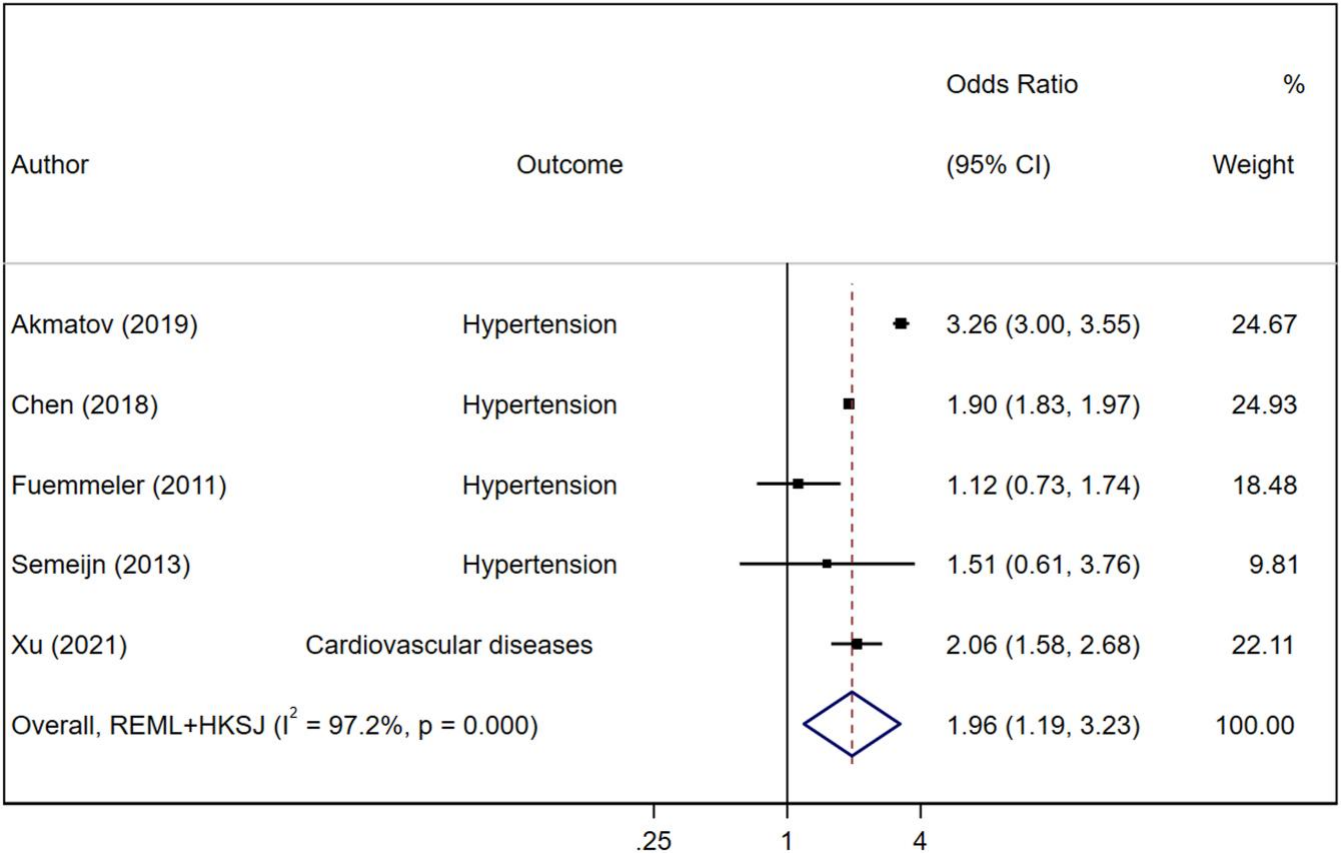
Forest plot of all studies describing associations between Attention‐deficit/hyperactivity disorder (ADHD) and cardiovascular diseases (CVDs) with adjusted estimates.

In the main analysis, we first explored the associations between ADHD and CVDs among seven studies that provided adjusted effect sizes (Akmatov et al., 2019; Chen et al., 2018; Du Rietz et al., 2021; Fuemmeler et al., 2011; Li et al., 2022; Ramos Olazagasti et al., 2013; Xu et al., 2022). The three Swedish register‐based studies (Chen et al., 2018; Du Rietz et al., 2021; Li et al., 2022) involved similar population, therefore only the largest one was included in the main analysis (Chen et al., 2018). A total of 8,196,648 (ADHD = 332,619) individuals from five studies were included in the main meta‐analysis of adjusted estimates. We found that ADHD was associated with a significantly increased risk of CVDs (pooled OR = 1.96; 95% CI = 1.19–2.23) (Figure 3). However, heterogeneity was high and significant (*Q* = 140.74, *P*_Q_ < 0.001, *I* ^2^ = 97.2%) and the between‐study standard variance was not high (tau^2^ is 0.09, 95%CI = 0.03–0.44) but with wide CI. The effect size was robust in the leave‐one‐out sensitivity analysis (Figure S1), and the effect size was not driven by one single study. There was no evidence of publication bias for the primary outcomes (Egger test: *p* = 0.81) (Figures S2 and S3).

When we repeated the analysis among six studies (Akmatov et al., 2019; Fuemmeler et al., 2011; Grisaru et al., 2018; Nilgün et al., 2018; Ramos Olazagasti et al., 2013; Spencer et al., 2014) with unadjusted associations (five of six estimates were manually calculated), a positive association was found for CVDs in individuals with ADHD compared to controls (OR = 1.48, 95% CI = 0.97–2.26; *Q* = 188.57, *P*_Q_ < 0.001, *I* ^2^ = 97.3%) but this was not statistically significant (Figure S4).

Table 3 summarizes the results from subgroup and sensitivity analyses based on adjusted estimates. First, in the analysis limited to adults, the association between ADHD and CVDs was significant among six studies (pooled OR = 1.73; 95% CI = 1.14–2.62) without significant heterogeneity (*Q* = 6.28, *P*_Q_ = 0.10, *I* ^2^ = 6.28%), while the only study in children reported a stronger association between ADHD and CVD (hypertension as main outcome) than adults (OR = 3.26; 95% CI = 3.00–3.55; Q_between‐group_ = 86.74, *p* < 0.0001). As age was a main factor affecting the heterogeneity of the results, we restricted the following sensitivity analyses on type of CVDs and data sources among adults. Second, among four studies with adjusted data on specific types of CVDs, we found that ADHD was associated with a higher risk of hypertension (OR = 1.55; 95% CI = 0.75–3.21, *Q* = 5.89, *P*_Q_ = 0.06, *I* ^2^ = 66.0%) and other CVDs (OR = 1.73; 95% CI = 1.19–2.68, *Q* = 3.20, *P*_Q_ = 0.20, *I* ^2^ = 37.7%), even though the association between ADHD and Hypertension was not statistically significant.

**TABLE 3:** Summary of results of subgroups analyses.

Study	Number of studies	Pooled ORs	Q	*P*_Q_	*I* ^2^
Adults	4	1.73 (1.14–2.62)	6.28	0.1	6.28%
Children	1	3.26 (3.00–3.55)	N/A	N/A	N/A
Adults:					
Hypertension	3	1.55 (0.75–3.21)	5.89	0.06	66.0%
Other CVDs	3	1.73 (1.19–2.68)	3.2	0.201	37.7%

Abbreviation: *P*_Q_, *p*‐value associated to the Q statistic of heterogeneity.

## DISCUSSION

The meta‐analysis provided evidence that ADHD was significantly associated with an increased risk of CVDs, after adjusting for potential confounders. However, due to the limited number of available studies and lack of information in the original studies, it is currently unclear whether these associations can be explained by other confounders. Therefore, there is a critical need for future studies on this understudied topic to advance the understanding of the underlying mechanism for the observed association between ADHD and CVDs, especially the role of ADHD medications and other mediating factors, such as mental comorbid conditions (i.e. substance use disorders or depression). A better understanding of major mediating factors may inform clinical guidelines about how to intervene on CVDs in ADHD.

Compared with evidence from meta‐analysis for well‐established risk factors for CVDs (i.e., smoking, obesity, physical activity/sedentary behavior, diabetes, dyslipidemia and sleep disorders) among adults, the observed magnitude of association between ADHD and CVDs was slightly smaller than the magnitude of association for sedentary behavior (Wilmot et al., 2012), diabetes (Garcia‐Argibay et al., 2023), and smoking (Mons et al., 2015), but stronger than the magnitude for dyslipidemia (Sniderman et al., 2011), obesity (Dwivedi et al., 2020) and sleep disorders (Li et al., 2014) (Table 4). When comparing with other psychiatric disorders, the observed strength of associations between ADHD and CVDs is largely similar to estimates of associations of schizophrenia (Lambert et al., 2022) and substance use disorders (Gan et al., 2021) with CVDs, but stronger than stress‐related disorder (Song et al., 2019), depression (Emdin et al., 2016), bipolar disorder (Lambert et al., 2022), and anxiety disorders (Table 4). (Batelaan et al., 2016) Therefore, it is important to call for enhanced clinical awareness of cardiovascular risk among adults with ADHD.

**TABLE 4:** The magnitude of associations between Attention‐deficit/hyperactivity disorder (ADHD), well‐established risk factors for cardiovascular diseases (CVDs), and psychiatric disorders with CVDs among adults.

	OR/HR/RR (95% CI)	Type of evidence
ADHD	1.73 (1.14–2.62)	Meta‐analysis of observational studies
Well‐established risk factors for CVD		
Sedentary behavior (Wilmot et al., 2012)	2.47 (1.44–4.24)	Meta‐analysis of observational studies
Diabetes (Garcia‐Argibay et al., 2023)	2.29 (1.48–3.55)	Meta‐analysis of observational studies
Smoking (Mons et al., 2015)	2.07 (1.82–2.36)	Meta‐analysis of observational studies
Dyslipidemia (Sniderman et al., 2011)	1.43 (1.35–1.51)	Meta‐analysis of observational studies
Obesity (Dwivedi et al., 2020)	1.43 (1.33–1.54)	Meta‐analysis of observational studies
Sleep disorders (Li et al., 2014)	1.33 (1.13–1.57)	Meta‐analysis of observational studies
Psychiatry comorbidities		
Schizophrenia (Lambert et al., 2022)	1.91 (1.52–2.41)	Meta‐analysis of observational studies
Substance use disorder (Gan et al., 2021)	1.70 (1.6 ˗ 1.9)	Cohort study
Stress‐related disorders (Song et al., 2019)	1.64 (1.45–1.84)	Cohort study
Depression (Emdin et al., 2016)	1.64 (0.84, 3.19)	Meta‐analysis of observational studies
Bipolar disorder (Lambert et al., 2022)	1.61 (1.34–1.94)	Meta‐analysis of observational studies
Anxiety disorders (Batelaan et al., 2016)	1.52 (1.36–1.71)	Meta‐analysis of observational studies

In the original studies, the type and number of possible confounding factors adjusted for varied across studies, and few studies provided a clear rationale for their covariate selection, which is a common problem in contemporary observational research on mental health (Larsson, 2022). Therefore, the significant adjusted association should also be interpreted with caution. In addition, inadequate adjustment for confounding was found in most studies (e.g., only four studies (Akmatov et al., 2019; Fuemmeler et al., 2011; Li et al., 2022; Xu et al., 2022) considered other potential confounders except for age and sex), and only one of the included studies adjusted for ADHD medication (Li et al., 2022), which may contribute to increased risk of CVDs (Habel et al., 2011; Levin et al., 2018). Only one of the previous studies used a sibling comparison design (Du Rietz et al., 2021) and suggested that the observed associations could also be partly explained by shared familial factors. Therefore, it is currently unclear whether any of the observed association reflect a causal effect or confounding. Clearly, more research using different designs, such as within‐family analysis or instrumental variable design, are needed to systematically adjust for a broad set of possible confounders and further explore the underlying mechanisms. Support for a potential causal association of ADHD with coronary heart disease (Leppert et al., 2021) and stroke (Du et al., 2022) has been observed in a recent Mendelian Randomization study. In addition, to identify critical intervention targets, more research is also needed on potential mediating factors, such as ADHD medication use, other psychiatric comorbidities and metabolic diseases.

Heterogeneity was high and significant for the main analyses, indicating that the pooled OR cannot appropriately summarize results from all the included individuals' studies, but it has limited effect on the conclusion of a positive association between ADHD and CVDs (Cortese et al., 2018; Imrey, 2020). When we restricted the analyses to different age group (children and adults), the associations remained stable, but the degree of heterogeneity substantially decreased to null, suggesting that age might be an important factor affecting the heterogeneity of the results.

Using subgroup meta‐analyses, we clarified the differences in associations between ADHD and different types of CVD outcomes. The available studies indicate that individuals with ADHD had a 73% higher risk of a broad range of CVDs than those without ADHD, and the strength of the associations was more pronounced for cardiac arrest, hemorrhagic stroke, and peripheral vascular disease/arteriosclerosis (Li et al., 2022). However, research from independent samples is needed to replicate their findings. On the other hand, previous studies indicating that ADHD‐related traits such as impulsivity, hostility, and time urgency/impatience, are associated with increased risk for hypertension (Fuemmeler et al., 2011; Yan et al., 2003). Consistently, we also found a potential elevated risk of hypertension among adults with ADHD, but this was not statistically significant. Therefore, more studies are needed to further explore the associations between ADHD and specific types of CVDs.

### Strengths and limitations

This is the first systematic review and meta‐analysis, with 421,224 individuals having ADHD, to assess the relationship between ADHD and CVDs. Additionally, we also conducted sensitivity and subgroup analyses to further evaluate the findings from the main analyses. However, our results should be considered in the context of some limitations. First, we reported unadjusted ORs as secondary outcomes, but most (83.3%) of the unadjusted ORs were from manually calculated effect sizes based on available information in the original studies, as these studies did not generate unadjusted effect size of the association between ADHD and CVDs. Second, we attempted to reduce publication bias by including both published and unpublished studies, but bias cannot be ruled out completely. Third, most of the included studies were conducted in Europe and the U.S., which limits the generalizability of the findings to other populations across the world. Therefore, more studies are needed to examine the associations between ADHD and CVDs using samples from different settings and regions. Fourth, the definition and measurements of ADHD and CVDs from original studies varied substantially. Due to the limited number of included studies, it is not possible to assess the associations among different definitions, which need to be evaluated in future systematic reviews and meta‐analyses on this topic.

### Future perspective

Compared with other psychiatric disorders (e.g., schizophrenia, major depression), the risk of CVDs in individuals with ADHD is largely understudied. A number of important research questions, therefore, need to be addressed in future research. First, except for studies with a specific focus on hypertension, the most commonly used measure of CVDs was a broad category, encompassing a wide range of circulatory system diseases. Future studies are needed to explore the associations between ADHD and specific types of CVDs, which is critical to enabling risk reductions via targeted intervention and preventative efforts. Second, future research should also consider careful adjustments for a wide range of possible confounders of the observed associations between ADHD and CVDs, including but not limited to early life risk factors (e.g., preterm birth (Bavineni et al., 2019; Franz et al., 2018) and birth weight (Franz et al., 2018; Mohseni et al., 2020)) and socioeconomic status (Clark et al., 2009; Russell et al., 2016), as well as unmeasured familial factors (Du Rietz et al., 2021). To further identify critical intervention targets, future research also needs to explore the role of potential mediating factors, such as smoking (Ambrose & Barua, 2004; McClernon & Kollins, 2008), sleep problems (Fan et al., 2019; Owens, 2005), metabolic conditions (Landau & Pinhas‐Hamiel, 2019) (e.g., obesity (Powell‐Wiley et al., 2021), types 2 diabetes mellitus (Einarson et al., 2018), dyslipidemia (Hedayatnia et al., 2020), and in particular obesity given that a recent Mendelian Randomization study suggested that obesity may mediate the causal association between ADHD and coronary heart disease (Leppert et al., 2021). Third, only one study has explored the role of psychiatric comorbidities and the use of psychotropic medications in the studied associations. This is an important limitation given that ADHD is frequently comorbid with other psychiatric disorders (e.g., mood disorder and substance use disorder), and these conditions and related medications are in turn associated with increased risk of CVDs (Li et al., 2022; Vancampfort et al., 2015). Future studies should examine to what extent the observed associations between ADHD and CVDs could be explained by ADHD medications, other psychiatric disorders and related medications. Fourth, research is also needed to test the potential sex‐ and age differences in the associations of ADHD with CVDs, which is helpful for risk stratification and individualized treatment recommendations in clinical practice. Taken together, more studies on this topic are needed, especially using different study designs, such as matched‐cohort studies (Du Rietz et al., 2021; Fuemmeler et al., 2011), genetically‐informed studies (e.g., sibling comparison studies and Mendelian Randomization studies) (Leppert et al., 2021) and advanced statistical methods (e.g. propensity score methods) to account for confounding.

## CONCLUSION

This systematic review and meta‐analysis suggest a significant positive association between ADHD and CVDs. More efforts are needed to this substantially understudied research field. In particular, mediation effects by psychiatric comorbidities and related medications, as well as the causal mechanisms underlying the association, deserve further attention because of their important public health implications.

## AUTHOR CONTRIBUTIONS

**Lin Li:** Conceptualization, Data curation, Formal analysis, Methodology, Software, Validation, Visualization, Writing – original draft, Writing – review & editing. **Honghui Yao:** Data curation, Methodology, Writing – review & editing. **Le Zhang:** Data curation, Methodology, Writing – review & editing. **Miguel Garcia‐Argibay:** Conceptualization, Data curation, Methodology, Writing – review & editing. **Ebba Du Rietz:** Funding acquisition, Writing – review & editing. **Marco Solmi:** Methodology, Writing – review & editing. **Samuele Cortese:** Methodology, Writing – review & editing. **J. Antoni Ramos‐Quiroga:** Methodology, Writing – review & editing. **Marta Ribasés**: Methodology, Writing – review & editing. **Zheng Chang:** Conceptualization, Funding acquisition, Software, Supervision, Writing – review & editing. **Henrik Larsson:** Conceptualization, Funding acquisition, Supervision, Writing – review & editing.

## CONFLICTS OF INTEREST STATEMENT

Dr Larsson has served as a speaker for Medice, Evolan Pharma and Shire/Takeda and has received research grants from Shire/Takeda; all outside the submitted work. He is Editor‐in‐Chief of JCPP Advances. Ebba Du Rietz has served as a speaker for Shire Sweden AB outside the submitted work. Dr. Solmi received honoraria/has been a consultant for Angelini, Lundbeck, Otsuka. He is a joint editor of JCPP Advances. S Cortese declares honoraria and reimbursement for travel and accommodation expenses for lectures from the following non‐profit associations: Association for Child and Adolescent Central Health, Canadian ADHD Alliance Resource, British Association of Pharmacology, and from Healthcare Convention for educational activity on ADHD. He serves on the Editorial Advisory Board for JCPP Advances. Dr. J. Antoni was on the speakers' bureau and/or acted as consultant for Janssen‐Cilag, Novartis, Shire, Takeda, Bial, Shionogi, Sincrolab, Novartis, BMS, Medice, Rubió, Uriach, Technofarma and Raffo in the last 3 years. He also received travel awards (air tickets + hotel) for taking part in psychiatric meetings from Janssen‐Cilag, Rubió, Shire, Takeda, Shionogi, Bial and Medice. The Department of Psychiatry chaired by him received unrestricted educational and research support from the following companies in the last 3 years: Janssen‐ Cilag, Shire, Oryzon, Roche, Psious, and Rubió. No other disclosures were reported. Z Chang serves on the Editorial Advisory Board for JCPP Advances.

### OPEN RESEARCH BADGES

This article has earned a Preregistered Research Designs badge for having a preregistered research design, available at (https://www.crd.york.ac.uk/prospero/display_record.php?RecordID=274367).

## ETHICAL CONSIDERATIONS

Not applicable to this research review.

## Supporting information

Supplementary Material

## Data Availability

Not applicable to this research review.
