# Supplementary material for: Attention‐deficit/hyperactivity disorder is associated with increased risk of cardiovascular diseases: A systematic review and meta‐analysis
Source: JCPP Adv. 2023 Apr 5;3(3):e12158. doi: 10.1002/jcv2.12158 (PMC10501695; doi:10.1002/jcv2.12158)
Supplement: Supplementary file 1 — Supplementary Material [file JCV2-3-e12158-s001.docx]

**Supporting Information**

**Table S1:** Search strategy and results from each electronic database

**Table S2:** Studies excluded from the systematic review after full-text screen, with reasons

**Table S3:** Quality assessment by Newcastle-Ottawa Scale

**Figure S1** Results of leave-one-out sensitivity analysis. The vertical axis shows the omitted study. Every circle indicates the pooled OR when the left study is omitted in this meta-analysis. The two ends of every broken line represent the respective 95% confidence interval

**Figure S2.** Results from Egger's test for small study effect, suggested there was no small study effects (P=0.67)

**Figure S3.** Publication bias of included studies (those with adjusted estimates)

**Figure S4.** Forest plot of all studies describing associations between ADHD and CVDs with crude estimates

**Table S1** Search strategy and results from each electronic database

| Electronic database | Search terms | Limits | Results |
| --- | --- | --- | --- |
| PubMed | ("cardiovascular diseases"[MeSH Terms] OR "cardiovascular system"[MeSH Terms] OR "coronary disease"[MeSH Terms] OR "heart diseases"[MeSH Terms] OR "death, sudden"[MeSH Terms] OR "death, sudden, cardiac"[MeSH Terms] OR "arrhythmias, cardiac"[MeSH Terms] OR "tachycardia"[MeSH Terms] OR "myocardial infarction"[MeSH Terms] OR "hypertension"[MeSH Terms] OR ("myocardial ischemia"[MeSH Terms] OR "coronary artery disease"[MeSH Terms]) OR "coronary artery disease"[MeSH Terms] OR "heart failure"[MeSH Terms] OR "heart arrest"[MeSH Terms] OR "myocarditis"[MeSH Terms] OR "angina pectoris"[MeSH Terms] OR "cardiomyopathies"[MeSH Terms] OR "peripheral arterial disease"[MeSH Terms] OR "ischemic attack, transient"[MeSH Terms] OR "cerebrovascular disorders"[MeSH Terms] OR (("cerebrum"[MeSH Terms] OR "brain"[MeSH Terms]) AND "vascular diseases"[MeSH Terms]) OR "stroke"[MeSH Terms] OR "cardiovascular disease"[Title/Abstract] OR "cardiovascular diseases"[Title/Abstract] OR "cardiovascular event"[Title/Abstract] OR "cardiovascular events"[Title/Abstract] OR "cardiovascular disorder"[Title/Abstract] OR "cardiovascular disorders"[Title/Abstract] OR "coronary heart disease"[Title/Abstract] OR "coronary heart diseases"[Title/Abstract] OR "heart diseases"[Title/Abstract] OR "heart disease"[Title/Abstract] OR "sudden death"[Title/Abstract] OR "sudden cardiac death"[Title/Abstract] OR "arrhythmia"[Title/Abstract] OR "tachycardia"[Title/Abstract] OR "tachyarrhythmia"[Title/Abstract] OR "myocardial infarction"[Title/Abstract] OR "heart attack"[Title/Abstract] OR "hypertension"[Title/Abstract] OR "hypertensive"[Title/Abstract] OR "ischemic heart disease"[Title/Abstract] OR "heart failure"[Title/Abstract] OR "cardiac arrest"[Title/Abstract] OR "myocarditis"[Title/Abstract] OR "angina"[Title/Abstract] OR "cardiomyopathy"[Title/Abstract] OR "peripheral artery disease"[Title/Abstract] OR "peripheral artery diseases"[Title/Abstract] OR "transient ischemic attack"[Title/Abstract] OR "transient ischemic attacks"[Title/Abstract] OR "transient ischaemic attack"[Title/Abstract] OR "transient ischaemic attacks"[Title/Abstract] OR "cerebrovascular disease"[Title/Abstract] OR "cerebrovascular diseases"[Title/Abstract] OR "cerebro vascular disease"[Title/Abstract] OR "cerebro vascular diseases"[Title/Abstract] OR "cerebral vascular disease"[Title/Abstract] OR "cerebral vascular diseases"[Title/Abstract] OR "stroke"[Title/Abstract]) AND ("attention deficit disorder with hyperactivity"[MeSH Terms] OR "central nervous system stimulants"[MeSH Terms] OR "methylphenidate"[MeSH Terms] OR "dexmethylphenidate hydrochloride"[MeSH Terms] OR "methamphetamine"[MeSH Terms] OR "dextroamphetamine"[MeSH Terms] OR "amphetamine"[MeSH Terms] OR "amphetamines"[MeSH Terms] OR "lisdexamfetamine dimesylate"[MeSH Terms] OR "atomoxetine hydrochloride"[MeSH Terms] OR "guanfacine"[MeSH Terms] OR "clonidine"[MeSH Terms] OR "viloxazine"[MeSH Terms] OR "ADHD"[Title/Abstract] OR "attention deficit hyperactivity disorder"[Title/Abstract] OR "attention-deficit"[Title/Abstract] OR "hyperkinetic disorder"[Title/Abstract] OR "hyperkinetic syndrome"[Title/Abstract] OR "psychostimulant"[Title/Abstract] OR "psychostimulants"[Title/Abstract] OR "central nervous system stimulant"[Title/Abstract] OR "central nervous system stimulants"[Title/Abstract] OR "stimulant"[Title/Abstract] OR "stimulants"[Title/Abstract] OR "non-stimulant"[Title/Abstract] OR "non-stimulants"[Title/Abstract] OR "methylphenidate"[Title/Abstract] OR "dexmethylphenidate"[Title/Abstract] OR "methamphetamine"[Title/Abstract] OR "dextroamphetamine"[Title/Abstract] OR "amphetamine"[Title/Abstract] OR "amphetamines"[Title/Abstract] OR "lisdexamfetamine"[Title/Abstract] OR "atomoxetine"[Title/Abstract] OR "guanfacine"[Title/Abstract] OR "clonidine"[Title/Abstract] OR "viloxazine"[Title/Abstract]) AND ("epidemiology"[MeSH Terms] OR "cohort studies"[MeSH Terms] OR "case control studies"[MeSH Terms] OR "longitudinal studies"[MeSH Terms] OR "retrospective studies"[MeSH Terms] OR "population"[MeSH Terms] OR "population groups"[MeSH Terms] OR "registries"[MeSH Terms] OR "records"[MeSH Terms] OR "epidemiolog*"[Title/Abstract] OR "observational"[Title/Abstract] OR "cohort"[Title/Abstract] OR "case-control"[Title/Abstract] OR "case-control"[Title/Abstract] OR "follow-up"[Title/Abstract] OR "follow-up"[Title/Abstract] OR "longitudinal"[Title/Abstract] OR "prospective"[Title/Abstract] OR "retrospective"[Title/Abstract] OR "population*"[Title/Abstract] OR "regist*"[Title/Abstract] OR "claims"[Title/Abstract] OR "record"[Title/Abstract]) | Abstract/Title | 1978 hits |
| Embase and Medline | #1 'cardiovascular disease':ab,ti OR 'cardiovascular diseases':ab,ti OR 'cardiovascular event':ab,ti OR 'cardiovascular events':ab,ti OR 'cardiovascular disorder':ab,ti OR 'cardiovascular disorders':ab,ti OR 'coronary heart disease':ab,ti OR 'coronary heart diseases':ab,ti OR 'heart diseases':ab,ti OR 'heart disease':ab,ti OR 'sudden death':ab,ti OR 'sudden cardiac death':ab,ti OR arrhythmia:ab,ti OR tachycardia:ab,ti OR tachyarrhythmia:ab,ti OR 'myocardial infarction':ab,ti OR 'heart attack':ab,ti OR hypertension:ab,ti OR hypertensive:ab,ti OR 'ischemic heart disease':ab,ti OR 'heart failure':ab,ti OR 'cardiac arrest':ab,ti OR myocarditis:ab,ti OR angina:ab,ti OR cardiomyopathy:ab,ti OR 'peripheral artery disease':ab,ti OR 'peripheral artery diseases':ab,ti OR 'transient ischemic attack':ab,ti OR 'transient ischemic attacks':ab,ti OR 'transient ischaemic attack':ab,ti OR 'transient ischaemic attacks':ab,ti OR 'cerebrovascular disease':ab,ti OR 'cerebrovascular diseases':ab,ti OR 'cerebro vascular disease':ab,ti OR 'cerebro vascular diseases':ab,ti OR 'cerebral vascular disease':ab,ti OR 'cerebral vascular diseases':ab,ti OR stroke:ab,ti  #2 adhd:ab,ti OR 'attention-deficit hyperactivity disorder':ab,ti OR 'attention deficit':ab,ti OR 'hyperkinetic disorder':ab,ti OR 'hyperkinetic syndrome':ab,ti OR psychostimulant:ab,ti OR psychostimulants:ab,ti OR 'central nervous system stimulant':ab,ti OR 'central nervous system stimulants':ab,ti OR stimulant:ab,ti OR stimulants:ab,ti OR 'non stimulant':ab,ti OR 'non stimulants':ab,ti OR methylphenidate:ab,ti OR dexmethylphenidate:ab,ti OR methamphetamine:ab,ti OR dextroamphetamine:ab,ti OR amphetamine:ab,ti OR amphetamines:ab,ti OR lisdexamfetamine:ab,ti OR atomoxetine:ab,ti OR guanfacine:ab,ti OR clonidine:ab,ti OR viloxazine:ab,ti  #3 epidemiolog*:ab,ti OR observational:ab,ti OR cohort:ab,ti OR 'case control':ab,ti OR 'case-control':ab,ti OR 'follow up':ab,ti OR 'follow-up':ab,ti OR longitudinal:ab,ti OR prospective:ab,ti OR retrospective:ab,ti OR population*:ab,ti OR regist*:ab,ti OR claims:ab,ti OR record:ab,ti  #1 AND #2 AND #3 | None | 1825 hits |
| PscyINFO | 1. (cardiovascular disease or cardiovascular diseases or cardiovascular event or cardiovascular events or cardiovascular disorder or cardiovascular disorders or coronary heart disease or coronary heart diseases or heart diseases or heart disease or sudden death or sudden cardiac death or arrhythmia or tachycardia or tachyarrhythmia or myocardial infarction or heart attack or hypertension or hypertensive or ischemic heart disease or heart failure or cardiac arrest or myocarditis or angina or cardiomyopathy or peripheral artery disease or peripheral artery diseases or transient ischemic attack or transient ischemic attacks or transient ischaemic attack or transient ischaemic attacks or cerebrovascular disease or cerebrovascular diseases or cerebro vascular disease or cerebro vascular diseases or cerebral vascular disease or cerebral vascular diseases or stroke).ab,ti.  2. (ADHD or attention-deficit hyperactivity disorder or attention deficit or hyperkinetic disorder or hyperkinetic syndrome or psychostimulant or psychostimulants or central nervous system stimulant or central nervous system stimulants or stimulant or stimulants or non-stimulant or non-stimulants or methylphenidate or dexmethylphenidate or methamphetamine or dextroamphetamine or amphetamine or amphetamines or lisdexamfetamine or atomoxetine or guanfacine or clonidine or viloxazine).ab,ti.  3. (epidemiolog* or observational or cohort or case control or case-control or follow up or follow-up or longitudinal or prospective or retrospective or population* or regist* or claims or record).ab,ti.  4. 1 and 2 and 3 | None | 1081 hits |
| Web of Science | TS=(cardiovascular disease OR cardiovascular diseases OR cardiovascular event OR cardiovascular events OR cardiovascular disorder OR cardiovascular disorders OR coronary heart disease OR coronary heart diseases OR heart diseases OR heart disease OR sudden death OR sudden cardiac death OR arrhythmia OR tachycardia OR tachyarrhythmia OR myocardial infarction OR heart attack OR hypertension OR hypertensive OR ischemic heart disease OR heart failure OR cardiac arrest OR myocarditis OR angina OR cardiomyopathy OR peripheral artery disease OR peripheral artery diseases OR transient ischemic attack OR transient ischemic attacks OR transient ischaemic attack OR transient ischaemic attacks OR cerebrovascular disease OR cerebrovascular diseases OR cerebro vascular disease OR cerebro vascular diseases OR cerebral vascular disease OR cerebral vascular diseases OR stroke) AND TS=(ADHD OR attention-deficit hyperactivity disorder OR attention deficit OR hyperkinetic disorder OR hyperkinetic syndrome OR psychostimulant OR psychostimulants OR central nervous system stimulant OR central nervous system stimulants OR stimulant OR stimulants OR non-stimulant OR non-stimulants OR methylphenidate OR dexmethylphenidate OR methamphetamine OR dextroamphetamine OR amphetamine OR amphetamines OR lisdexamfetamine OR atomoxetine OR guanfacine OR clonidine OR viloxazine) AND TS=(epidemiolog* OR observational OR cohort OR case control OR case-control OR follow up OR follow-up OR longitudinal OR prospective OR retrospective OR population* OR regist* OR claims OR record) | None | 2525 hits |

**Table S2** Studies excluded from the systematic review after full-text screen, with reasons

|  | Reference | Reason(s) for exclusion |
| --- | --- | --- |
| 1 | Adams, H. R., et al. (2010). Learning and attention problems among children with pediatric primary hypertension. Pediatrics, 126, e1425-1429. | Not related to our topic. It is mainly focus the risk of comorbid ADHD among children with and without hypertension |
| 2 | Hansen, E., et al. (2012). Prevalence of ADHD symptoms in patients with congenital heart disease. Pediatr Int, 54, 838-843. | Not related to our topic. It is mainly focus the risk of ADHD among children with and without CHD |
| 3 | Gonzalez VJ, Kimbro RT, Cutitta KE, Shabosky JC, Bilal MF, Penny DJ, Lopez KN. Mental Health Disorders in Children With Congenital Heart Disease. Pediatrics. 2021 Feb;147(2):e20201693. | Not related to our topic. It is mainly focus the risk of ADHD among children with and without CHD |
| 4 | Tesli, M., et al. (2020). Investigation of Shared Genetic Risk Between ADHD and Cardiometabolic Traits in a Norwegian Family Cohort Using Polygenic Transmission Disequilibrium Test. Biological Psychiatry, 87, S300-S300. | Not related to our topic. It is mainly focus the genetic association between ADHD and Cardio metabolic |
| 5 | Uy-Evanado, A., et al. (2019). Attention deficit disorder and risk of sudden cardiac death. Heart rhythm, 16, 209. | No available full text |
| 6 | Fuemmeler, B. F., et al. (2010). Obesity and stage II hypertension in early adulthood are associated with attention deficit/hyperactivity (ADHD) symptoms. Obesity Reviews, 11, 308. | Duplicate |
| 7 | Garcia-Argibay. et al. (2022). The role of ADHD genetic risk in mid-to-late life somatic health conditions. Transl Psychiatry 12, 152. | The definition of ADHD is based on polygenetic risk scores, which is not fulfilled our inclusion criteria |

**Table S3** Quality assessment by Newcastle-Ottawa Scale

| Study | Study Design | Selection | Comparability | Outcome | Total |
| --- | --- | --- | --- | --- | --- |
| Akmatov (2019) | Cross-sectional | *** | * | *** | 7 |
| Chen (2018) | Cross-sectional | ** | * | *** | 6 |
| Du Rietz (2021) | Cohort study | *** | ** | *** | 8 |
| Fuemmeler (2011) | Cohort study | *** | ** | ** | 7 |
| Grisaru (2018) | Case-control | * |  | * | 2 |
| Li (2022) | Cohort study | *** | *** | *** | 9 |
| Nilgün (2019) | Case control | * |  | ** | 3 |
| Olazagasti (2013) | Cohort study | * |  | ** | 3 |
| Semeijn (2013) | Cross-sectional | ** | * | ** | 5 |
| Spencer (2014) | Case-control | * | * | *** | 5 |
| Xu (2021) | Cross-sectional | ** | ** | * | 5 |

**Figure S1** Results of leave-one-out sensitivity analysis. The vertical axis shows the omitted study. Every circle indicates the pooled OR when the left study is omitted in this meta-analysis. The two ends of every broken line represent the respective 95% confidence interval

**
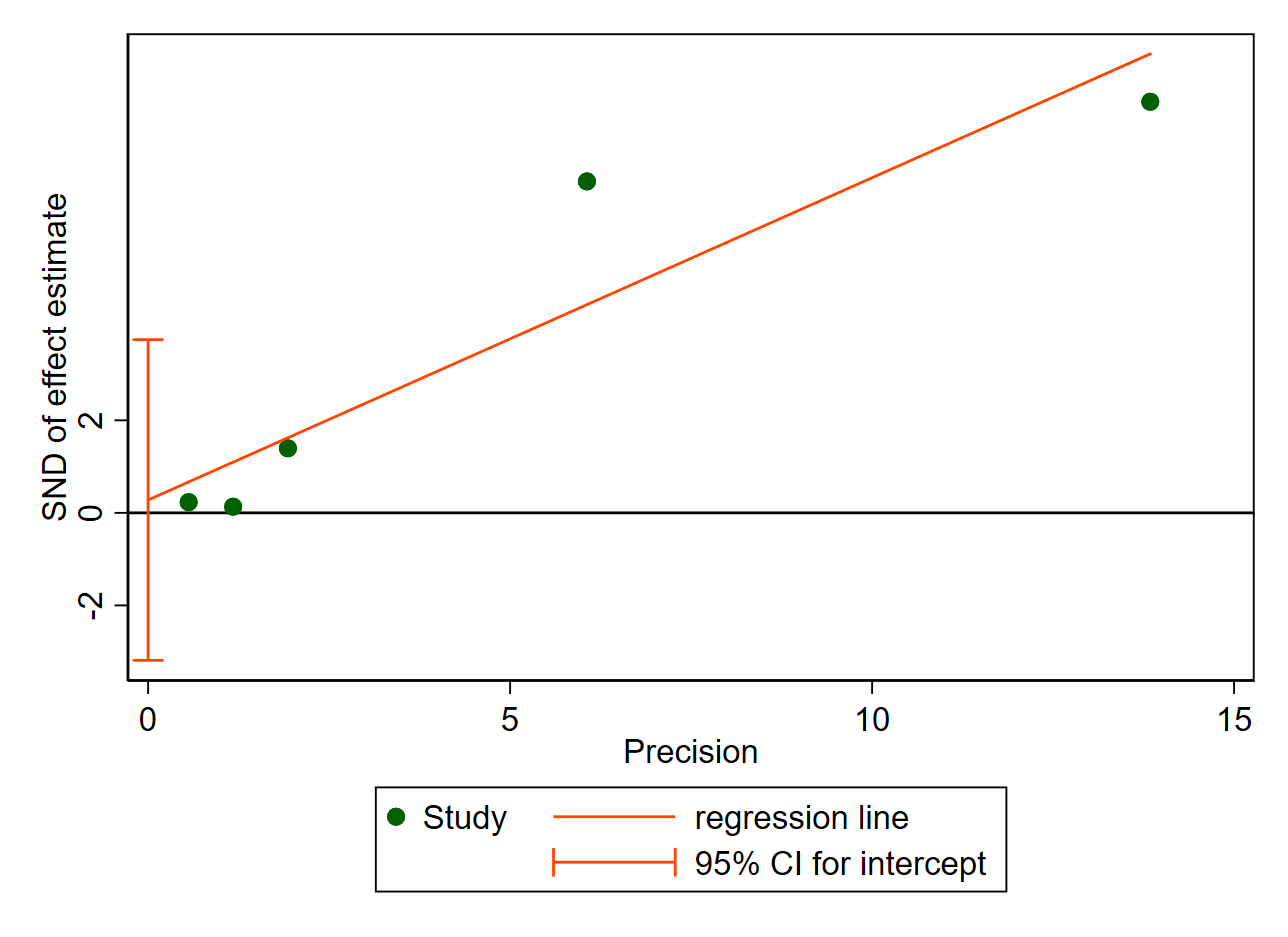
**

**Figure S2.** Results from Egger's test for small study effect, suggested there was no small study effects (P=0.67)

**
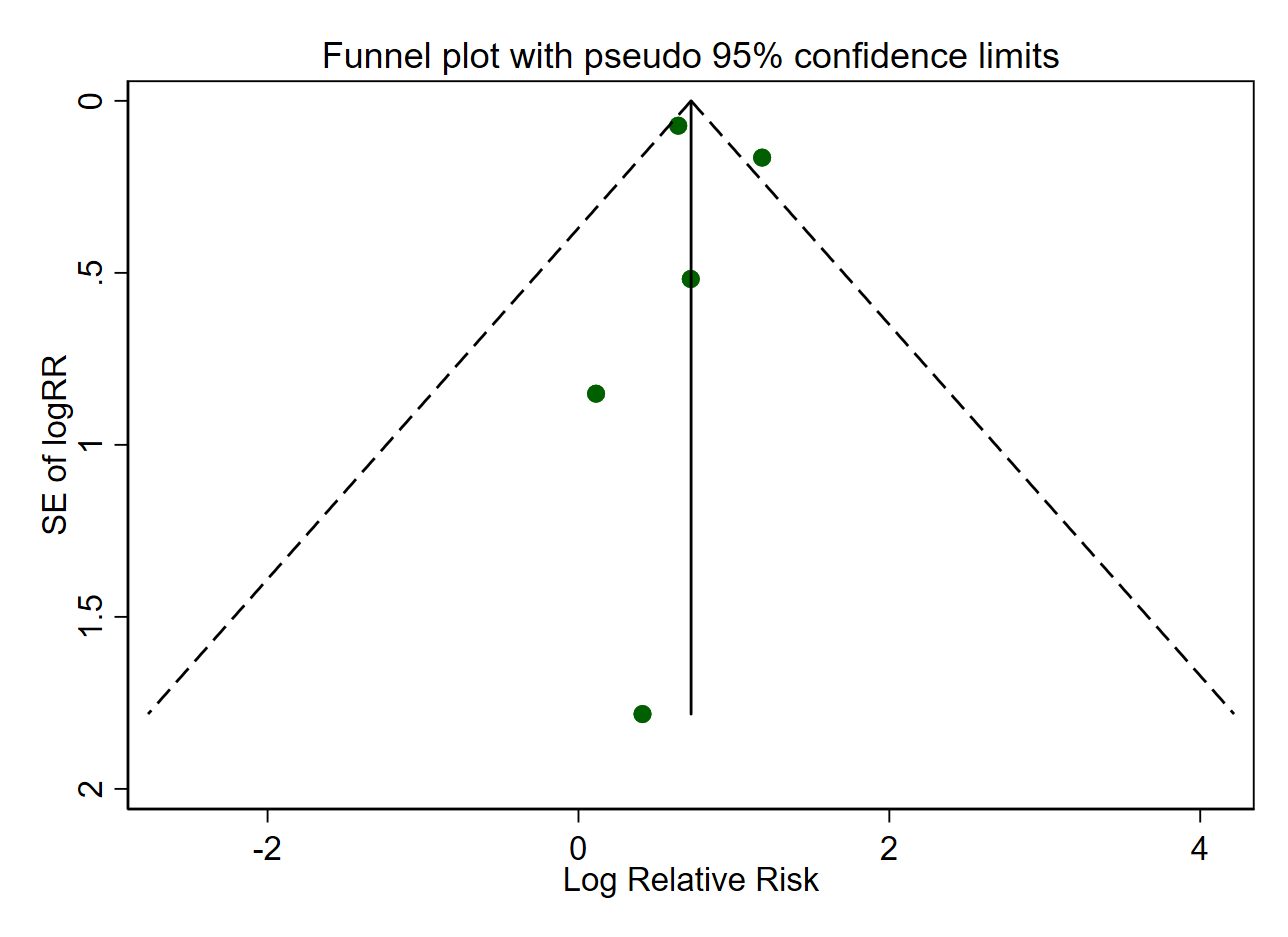
**

**Figure S3.** Publication bias of included studies (those with adjusted estimates)


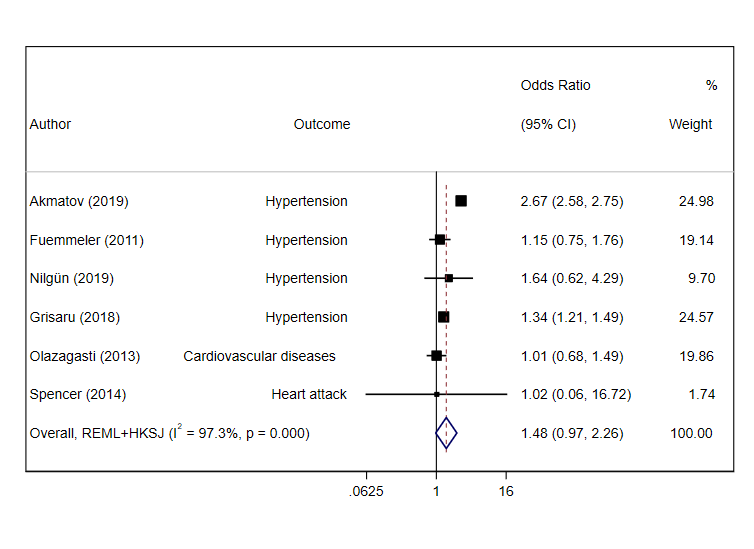


**Figure S4.** Forest plot of all studies describing associations between ADHD and CVDs with crude estimates
